# Effect of Transcranial Direct-Current Stimulation Combined with Treadmill Training on Balance and Functional Performance in Children with Cerebral Palsy: A Double-Blind Randomized Controlled Trial

**DOI:** 10.1371/journal.pone.0105777

**Published:** 2014-08-29

**Authors:** Natália de Almeida Carvalho Duarte, Luanda André Collange Grecco, Manuela Galli, Felipe Fregni, Cláudia Santos Oliveira

**Affiliations:** 1 Master Program in Rehabilitation Sciences, Movement Analysis Lab, University Nove de Julho, São Paulo, São Paulo, Brazil; 2 Doctoral Program in Rehabilitation Sciences, Movement Analysis Lab, University Nove de Julho, São Paulo, São Paulo, Brazil; 3 Dept. of Electronic Information and Bioengineering, Politecnico di Milano and IRCCS San Raffaele Pisana, Rome; 4 Laboratory of Neuromodulation & Center of Clinical Research Learning, Department of Physical Medicine & Rehabilitation, Spaulding Rehabilitation Hospital and Massachusetts General Hospital, Harvard Medical School, Boston, MA, United States of America; 5 Professor, Master and Doctoral Programs in Rehabilitation Sciences, Movement Analysis Lab, University Nove de Julho, São Paulo, São Paulo, Brazil; Earl and Christy Powell University, United States of America

## Abstract

**Background:**

Cerebral palsy refers to permanent, mutable motor development disorders stemming from a primary brain lesion, causing secondary musculoskeletal problems and limitations in activities of daily living. The aim of the present study was to determine the effects of gait training combined with transcranial direct-current stimulation over the primary motor cortex on balance and functional performance in children with cerebral palsy.

**Methods:**

A double-blind randomized controlled study was carried out with 24 children aged five to 12 years with cerebral palsy randomly allocated to two intervention groups (blocks of six and stratified based on GMFCS level (levels I-II or level III).The experimental group (12 children) was submitted to treadmill training and anodal stimulation of the primary motor cortex. The control group (12 children) was submitted to treadmill training and placebo transcranial direct-current stimulation. Training was performed in five weekly sessions for 2 weeks. Evaluations consisted of stabilometric analysis as well as the administration of the Pediatric Balance Scale and Pediatric Evaluation of Disability Inventory one week before the intervention, one week after the completion of the intervention and one month after the completion of the intervention. All patients and two examiners were blinded to the allocation of the children to the different groups.

**Results:**

The experimental group exhibited better results in comparison to the control group with regard to anteroposterior sway (eyes open and closed; p<0.05), mediolateral sway (eyes closed; p<0.05) and the Pediatric Balance Scale both one week and one month after the completion of the protocol.

**Conclusion:**

Gait training on a treadmill combined with anodal stimulation of the primary motor cortex led to improvements in static balance and functional performance in children with cerebral palsy.

**Trial Registration:**

Ensaiosclinicos.gov.br/RBR-9B5DH7

## Introduction

Cerebral palsy (CP) involves a set of neurophysiological impairments caused by a global reduction in subcortical activity that compromises the activity of corticospinal and somatosensory circuits [1], [2], [3], [4], [5], [6]. CP results in diminished activation of the central nervous system during the execution of movements [4]. A reduction in motor cortex excitability in children is associated with poor motor development [7]. Neurophysiological analyses have demonstrated global changes in cortex excitability in children with CP, even when the brain lesion is unilateral [8]. Such children have postural problems due to spasticity, muscle weakness and impaired muscle coordination. These postural problems can also affect motor development, leading to difficulties in performing basic functional actions, such as sitting, standing and walking [9], [10], [11], [12].

Adequate postural control involves a complex network of sensory and motor information. The integration of subcortical systems, such as the vestibular, sensorial and visual systems, is fundamental to the maintenance of balance. Moreover, posture is maintained through the combined efforts of the sensory motor cortex, supplementary motor area and pre-motor cortex [13].

While there is no cure for the brain lesion in CP, the manifestations of this condition can be minimized through neurorehabilitation [14]. Studies involving the administration of functional magnetic resonance on children with CP have demonstrated that rehabilitation resources are capable of promoting the activation of the primary motor cortex (M1) [14]. M1 is an important area of the brain that facilitates cerebral reorganization. Through a better understanding of the relationship between neuropathology and clinical function in CP, interventions can be individualized based on the neurological substrate available for recuperation, thereby maximizing the efficacy of the therapeutic process [15].

Recent studies have reported the benefits of gait training on a treadmill. Grecco *et al.* describe the positive effects of treadmill training in comparison to over-ground gait training on static and functional balance. The effects were found after 12 sessions of training at the aerobic threshold without body weight support. The benefits included an improvement in functional performance, suggesting that the motor effects can lead to greater independence in children with CP [10],[11].

Marchese *et al*. [16] suggest that repetitive sensory stimulation may favor the activation of important mechanisms that facilitate the motor learning process. Thus, like treadmill training, motor training may favor proprioceptive feedback, leading to adjustments for adequate postural balance and functional performance.

Transcranial direct-current stimulation (tDCS) is a safe, low-cost resource that can be used during motor therapy sessions and involves the administration of a weak electrical current to the scalp using sponge electrodes moistened with saline solution. The effects of stimulation are achieved by the movement of electrons due to electrical charges. The two poles are the anode (positive) and cathode (negative) electrodes. The electrical current flows from the positive pole to the negative pole, penetrating the skull and reaching the cortex, with different effects on biological tissues. Although most of the current is dissipated among the overlying tissues, a sufficient amount reaches the structures of the cortex and changes of membrane potential of the surrounding cells [17],[18]. tDCS is known to induce lasting changes in cortex excitability in both animals and humans. In rehabilitation processes, the aim of tDCS is to enhance local synaptic efficiency, thereby altering the maladaptive plasticity pattern that emerges following a cortex lesion. Stimulation is used to modulate the cortex activity by opening a pathway to increase and prolong functional gains achieved in physical therapy [19].

The authors believe that the combination of tDCS of the primary motor cortex and treadmill training can potentiate the effects on static balance. The hypothesis is that tDCS leads to the maintenance of the results following the interruption of the gait training protocol by inducing long-lasting changes in cortex excitability, thereby facilitating the learning process.

The aims of the present study were to determine the effects of tDCS applied over the primary motor cortex during ten sessions of treadmill gait training on balance and functional performance in children with PC and investigate whether the effects are maintained one month after the completion of the training sessions.

## Materials and Methods

### Ethics Statement

The protocol for this trial and supporting CONSORT checklist are available as supporting information; see Checklist S1 and Protocol S1. This study received approval from the Human Research Ethics Committee of the University Nove de Julho (Brazil) under process number 69803/2012 and was carried out in compliance with the ethical standards established by the Declaration of Helsinki. The study is registered with the Brazilian Registry of Clinical Trials under process number RBR-9B5DH7 (URL:http://www.ensaiosclinicos.gov.br/rg/RBR-9b5dh7/). There was a delay in releasing the record number for our study. To avoid delays in the conduct of the project or even loss of the sample, the recruitment of the sample was performed according to the previous schedule of the study. The authors confirm that all ongoing and related trials for this intervention are registered. All parents/guardians agreed to the participation of the children by signing a statement of informed consent.

### Design

**Figure 1:**
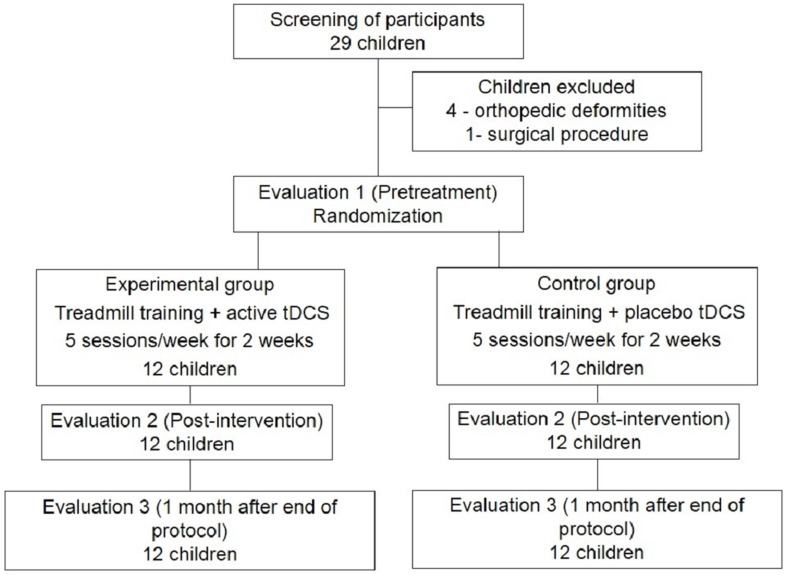
Flowchart of study based on Consolidated Standards of Reporting Trials.

Full details about the trial protocol have previously been reported [20] and can be found in the supplementary appendix, available at http://www.biomedcentral.com. A phase II, prospective, analytical, double-blind, randomized, placebo-controlled clinical trial was carried out. Figure 1 presents the CONSORT [21] flow chart of the study.

### Sample

The study took place at the Movement Analysis Lab, University Nove de Julho, Sao Paulo, Brazil, from November 2012 to September 2013. Twenty-nine children with CP were recruited from specialized outpatient clinics, from the physical therapy clinics of the University Nove de Julho and Center for Pediatric Neurosurgery, São Paulo, Brazil. The following were the inclusion criteria: diagnosis of spastic CP; classification on levels I, II or III of the Gross Motor Function Classification System (GMFCS); independent gait for at least 12 months; age between five and ten years; and degree of comprehension compatible with the execution of the procedures. The following were the exclusion criteria: history of surgery or neurolytic block in the previous 12 months; orthopedic deformities; epilepsy; metal implants in the skull or hearing aids.

All children who met the eligibility criteria (n = 24) were submitted to the initial evaluation and randomly allocated to an experimental group (treadmill training combined with active tDCS) and control group (treadmill training combined with placebo tDCS). Block randomization was used and stratified based on GMFCS level (levels I-II or level III). For each stratum, blocks of six were determined to minimize the risk of imbalance in the size of the separate samples. Numbered opaque envelopes were employed to ensure the concealment of the allocation. Each envelop contained a card stipulating to which group the child was allocated.

### Evaluation

All evaluation procedures were carried out by two examiners who were blinded to the allocation of the children to the different groups. All patients were blinded for this study. Evaluations consisted of stabilometric analysis as well as the administration of the Pediatric Balance Scale (PBS) and Pediatric Evaluation of Disability Inventory (PEDI) one week before the intervention (Evaluation 1), one week after the completion of the intervention (Evaluation 2) and one month after the completion of the intervention (Evaluation 3). Each evaluation was held on a single day. The child first rested in a chair for 20 minutes. The stabilometric analysis was then performed, followed by the PBS and then by the PEDI.

Stabilometric analysis was performed for the evaluation of static balance. For such, a force plate (Kistler model 9286BA) was used, which allows the record of oscillations of the center of pressure (COP). The acquisition frequency was 50 Hz, captured by four piezoelectric sensors positioned at the extremities of the force plate, which measured 40×60 cm. The data were recorded and interpreted using the SWAY software program (BTS Engineering), integrated and synchronized to the SMART-D 140 system. The child was instructed to remain in a standing position on the force plate, barefoot, arms alongside the body, with an unrestricted foot base, heels aligned and gazed fixed on a point marked at a distance of one meter at the height of the glabellum (adjusted for each child). Children classified on level III of the GMFCS used their normal gait-assistance device, which was positioned off the force plate. Thirty-second readings were taken under two conditions: eyes open and eyes closed. Displacement of the COP was measured in the anteroposterior (x axis) and mediolateral (Y axis) directions under each visual condition.

The PBS consists of 14 tasks resembling activities of daily living. The items are scored on a five-point scale ranging from 0 (inability to perform the activity without assistance) to 4 (ability to perform the activity independently). The maximum score is 56. Scoring is based on the time in which a position is maintained, the distance to which the upper limb is able to reach out in front of the body and the time required to complete the task [19].

The PEDI allows a quantitative evaluation of functional performance. This questionnaire is administered in interview format to one of the caregivers, who offers information on the child's performance on routine activities and typical tasks of daily living. The test is composed of three parts. The first part addresses abilities in the child's repertoire, which are grouped into three functional domains: self-care (73 items), mobility (59 items) and social function (65 items). Each item on this part receives a score of either 0 (child is unable to perform the activity) or 1 (activity is part of the child's repertoire). The score of each domain is determined by the sum of the items [22], [23].

### Intervention

One week after Evaluation 1, the children underwent the 10-session intervention protocol (5 weekly sessions for 2 weeks) involving treadmill training and tDCS (active or placebo). A specific test for children with CP was used to determine the treadmill training speed. This procedure was carried out based on the recommendations of Grecco *et al.* [9]. During the training sessions, the tDCS electrodes were positioned, the equipment was switched on and 20 minutes of gait training was performed simultaneously with anodal stimulation over the primary motor cortex (active or placebo). All children wore their normal braces during training, which were duly placed by the physiotherapist. Heart rate was monitored throughout the entire session to ensure an absence of overload on the cardiovascular system.

Gait training was performed on a treadmill (Inbramed, Millenium ATL, RS, Brazil). Two sessions were performed prior to the beginning of the protocol to familiarize the children with the treadmill. During these trial sessions, the children did not receive tDCS and treadmill speed was gradually increased based on the tolerance of each child. Training velocity was set at 80% of the maximum speed established during the exercise test [9].

Transcranial stimulation was applied with the tDCS Transcranial Stimulation device (Soterix Medical Inc., USA), using two sponge (non-metallic) electrodes (5×5 cm) moistened with saline solution. The anodal electrode was positioned over the primary motor cortex of the non-dominant hemisphere following the 10–20 International Electroencephalogram System [24] and the cathode was positioned in the supra-orbital region on the contralateral side.

To standardize the positioning of the electrodes of the diparetic patients, lower limb dominance was determined through self-reports; the children were asked: “Which leg is easier to move?” [25]. The anodal electrode was positioned over the hemisphere ispilateral to the dominant lower limb. Thus, the patients were stimulated in the more compromised hemisphere. In cases of hemiparesis, stimulation was standardized over the lesioned hemisphere.

In the experimental group, a 1-mA current was applied over the primary motor cortex for 20 minutes as the children performed the treadmill training. The device has a button that allows the operator to control the intensity of the current. In the first ten seconds, stimulation was gradually increased until reaching 1 mA and gradually diminished in the last ten seconds of the session. In the control group, the electrodes were positioned at the same sites and the device was switched on for 30 seconds, giving the children the initial sensation of the 1 mA current, but no stimulation was administered during the rest of the time. This is a valid control procedure in studies involving tDCS.

The number of sessions attended, maximum speed during treadmill training, duration of treadmill training and distance travelled in each session were recorded on the follow-up chart. Any problems or injuries that occurred during training were also recorded. All participants were instructed to maintain their routine daily activities.

### Statistical analysis

The sample size was calculated using the STATA 11 program and based on a study by Grecco *et al*. 2012 [9] [*Effect of treadmill training without partial weight support on functionality in children with cerebral palsy: Randomized controlled clinical trial*.] The PBS was selected as the primary outcome due to its proven validity and reliability in the literature for the evaluation of functional balance in children with CP and was therefore used in the sample size calculation. Based on a mean and standard deviation of 46.7±7.6 in the experimental group and 34.9±6.8 in the control group, 10 children in each group would be necessary for a bi-directional alpha of 0.05 and an 80% power. Twenty percent were added to each group to compensate for possible dropouts. Thus, the final sample was made up of 12 children in each group (total: 24 participants).

The Kolmogorov-Smirnov test was used to determine the adherence of the data to the Gaussian curve. The data proved to be parametric and were expressed as mean and standard deviation values. The effect size was calculated by the difference between means of the pre-intervention and post-intervention evaluations and was expressed with respective 95% confidence intervals. Repeated-measures ANOVA was used for the intra-group analyses and one-way ANOVA was used for the inter-group analyses. A p-value <0.05 was considered statistically significant. The data were organized and tabulated using the Statistical Package for the Social Sciences v.19.0 (SPSS, Chicago, IL, USA).

## Results

Twenty-nine children were screened and 24 were selected for participation in the present study, from November 2012 to September 2013. No losses occurred in either group. Table 1 displays the anthropometric characteristics and functional classification of the participants.

**Table 1:** Anthropometric characteristics and functional classification of children analyzed.

	Experimental group (n = 12)	Control group (n = 12)	
Age (years)*	7.8 (2.0)	8.1 (1.5)	p = 0.74
Body mass (Kg)*	27.9(2.5)	28.3(2.7)	p = 0.58
Height (cm)*	127.7(6.4)	128.2(7.4)	p = 0.73
Body mass index (Kg^2^/m)*	17.2(0.8)	17.8(1.5)	p = 0.39
GMFCS (I\II\III)**	(3\6\3)	(2\7\3)	p = 0.17
Topography (hemiparesis\diparesis)**	(3\9)	(2\10)	p = 0.77

Legend: GMFCS: Gross Motor Function Classification System.

*data expressed as mean (standard deviation); ** data representing frequency.

No statistically significant differences between groups were found regarding the anthropometric data, age or data referring to the primary or secondary outcomes at the baseline evaluation (p>0.05). Data as age, body mass, height and body mass index were analyzed by the independent t test. The GMFCS and topography was analyzed by the chi square test.

Table 2 displays the variables analyzed at baseline (Evaluation 1), after training (Evaluation 2) and at the follow up (Evaluation 3).

**Table 2:** Comparison of variables between experimental group and control group in three moments: Evaluation 1, Evaluation 2, and Evaluation 3.

	Experimental group			Control group		
	Evaluation 1	Evaluation 2	Evaluation 3	Evaluation 1	Evauation 2	Evaluation 3
PBS*	40.5(9.4)	45.3(7.9)	44.7(7.7)	39.1(9.8)	39.7(8.4)	39.5(9.3)
Oscillation AP EO*	18.6(3.9)	14.0(2.7)	14.2(2.6)	20.3(4.5)	15.8(3.6)	18.4(3.6)
Oscillation AP EC*	24.3(5.6)	17.1(4.3)	17.7(4.6)	24.2(4.8)	22.7(4.1)	23.2(4.1)
Oscillation ML EO*	20.3(4.5)	14.7(3.6)	15.3(4.1)	19.2(4.3)	18.6(3.2)	18.8(3.1)
Oscillation ML EC*	25.4(5.5)	18.9(4.3)	19.7(4.1)	25.1(5.2)	22.9(4.2)	22.8(3.6)
Self-care*	46.1(10.0)	48.0(9.5)	47.8(9.2)	45.0(9.2)	45.5(9.3)	45.6(9.4)
Mobility*	38.0(8.5)	41.7(7.4)	40.9(7.7)	38.3(7.4)	39.5(7.6)	38.8(7.0)

Legend: PBS (Pediatric Balance Scale), AP EO (Anteroposterior Eyes Open); AP EC (Anteroposterior Eyes Closed); ML EO (Mediolateral Eyes Open); ML EC (Mediolateral Eyes Closed).

*Data expressed as mean and standard deviation.

**Figure 2:**
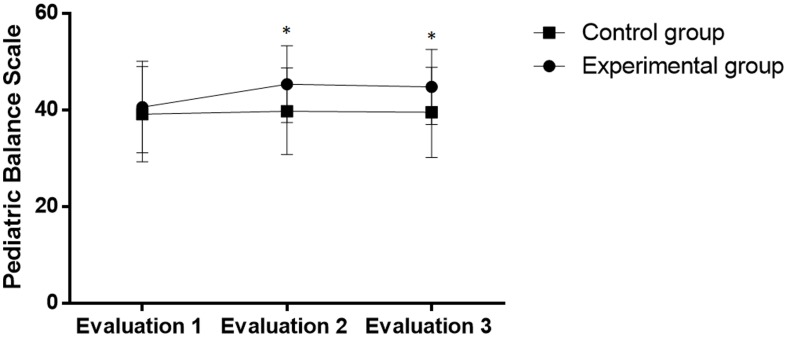
PBS scores in both groups before and after intervention. *statistically significant difference between groups (p<0.05).

The PBS was chosen as the primary outcome due to the fact that this scale allows the evaluation of functional balance and has proven validity for use on children with CP. The experimental group exhibited the effect of training with tDCS, as demonstrated by the increase in the final score after training and at the follow up evaluation [F (1.33) = 3.9; p = 0.05] (Figure 2). In contrast, no significant effect on the PBS score was found in the control group after the intervention [F (1.11) = 1.3; p = 0.27].

**Figure 3:**
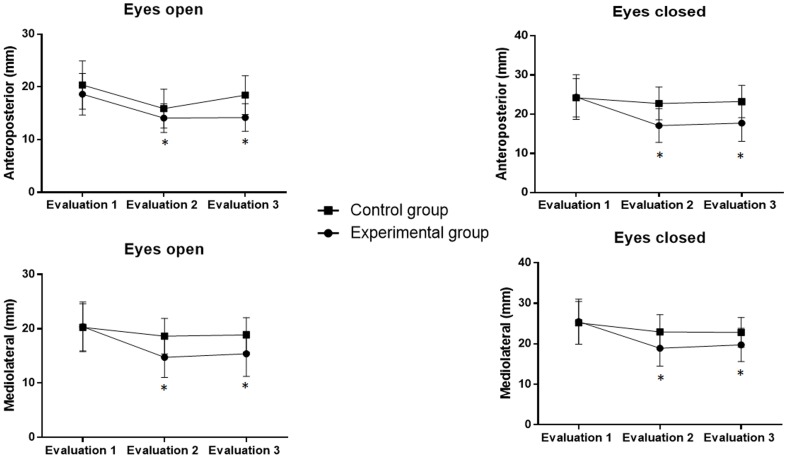
Oscillations of center of pressure. A) anteroposterior sway with eyes open; B) mediolateral sway with eyes open; C) anteroposterior sway with eyes closed; D) mediolateral sway with eyes closed. *Statistically significant difference between groups (p<0.05).

The stabilometric evaluation revealed positive effects on the reduction in body sway in the anteroposterior direction with eyes open [F (2.33) = 7.1; p = 0.002], anteroposterior direction with eyes closed [F (2.22) = 24.3; p<0.0001], mediolateral direction with eyes open [F (2.33) = 4.0; p = 0.02] and mediolateral direction with eyes closed [F (2.33) = 3.6; p = 0.03] (Figure 3). The experimental group maintained these effects on anteroposterior and mediolateral sway with eyes open and closed after the intervention (p<0.05). In the control group, the effect was maintained at Evaluation 3 only with regard to mediolateral sway with eyes closed [F (1.11) = 18.4; p = 0.001).

**Figure 4:**
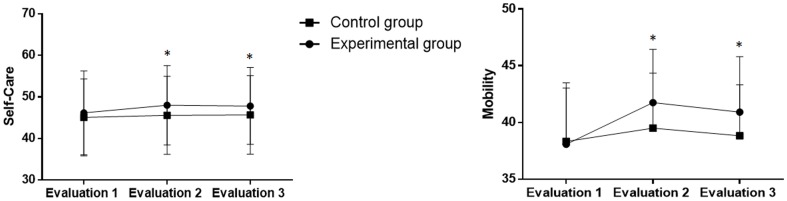
Self-care and mobility scores on PEDI in both groups before and after intervention.

Regarding the PEDI, an increase in the final score was found for the mobility [F (2.22) = 19.2; p<0.0001] and self-care [F (2.22) = 9.90; p = 0.0008] subscales. In the intra-group analysis, positive effects were found after treatment regarding self-care in both groups, but these effects were not maintained at Evaluation 3. Moreover, only the experimental group exhibited a positive effect regarding mobility after treatment (Evaluation 2). Figure 4 displays the PEDI results in both groups.

In the intra-group analysis, repeated-measures ANOVA revealed significant differences in both groups following motor training, with a reduction in oscillations of the COP one week after the end of the protocols. However, only the experimental group maintained this reduction one month after the protocol (Evaluation 3). The experimental group also exhibited improvements in regarding the balance scale. No significant intra-group differences were found with regard to self-care and functional mobility following treadmill training with tDCS.

In the inter-group analysis, one-way ANOVA revealed significant differences between groups. The experimental group exhibited significantly lower oscillations of the COP in the anteroposterior (experimental group with eyes open: 18.6±3.9, 14.0±2.7 and 14.2±1.9 mm; experimental group with eyes closed: 24.3±5.6, 17.1±4.3 and 17.7±4.6 mm; control group with eyes open: 20.3±4.5, 15.8±3.6 and 18.4±3.7 mm; control group with eyes closed: 24.2±4.8, 22.7±4.1 and 23.2±3.1 mm) and mediolateral (experimental group with eyes open: 20.3±4.5, 14.7±3.6 and 15.3±4.1 mm; experimental group with eyes closed: 25.4±18.9, 18.9±4.3 and 19.7±4.1 mm; control group with eyes open: 20.2±4.3, 18.6±3.2 and 18.8±3.1 mm; control group with eyes closed: 25.1±5.2, 22.9±4.2 and 22.8±3.6 mm) directions. These differences were found both one week and one month after the end of the interventions (Figure 2).

The experimental group also had better scores on the pediatric balance scale (experimental group: 40.5±9.4, 45.3±7.9 and 44.7±6.7; control group: 39.1±9.8, 39.7±8.9 and 39.5±9.3) (Figure 3).

However, no significant differences between groups were found regarding the self-care (experimental group: 46.1±10.8, 48.0±9.5 and 47.3±9.2; control group: 45.0±9.2, 45.5±9.3 and 45.6±8.9) or mobility (experimental group: 38.0±8.5, 41.7±7.4 and 40.9±7.7; control group: 39.3±7.4, 39.5±6.9 and 38.8±7.0) subscales of the PEDI (Figure 4).

## Discussion

There has been increasing use of tDCS in the rehabilitation of patients with lasting neurological effects following a brain lesion, especially in cases of stroke. Studies have also demonstrated the benefits of this technique in patients with Parkinson's disease, pain and depression. The method has proven to be promising and safe on adults [18].

Studies involving children also suggest that the method is safe, but requires lesser intensity of the electrical current. Through computations modeling, Minhas *et al*. (2012) [26] found that lesser intensity than that conventionally used on adults is capable of cortex stimulation in children. Based on the results achieved with stroke victims and studies that demonstrate an absence of adverse effects in children, the aim of the present investigation was to determine whether anodal stimulation of the primary motor cortex in the dominant hemisphere combined with treadmill training would lead to an increase in or the maintenance of the effect of treadmill training on static and functional balance in children with CP.

Previous studies have demonstrated that treadmill training without body support and at a speed determined by a prior exercise test leads to improvements in both static and functional balance and favors functional performance in children with CP [9]. In the present study, an established treadmill training protocol with effects demonstrated in the literature was used to determine whether tDCS is valid in children with CP classified on levels I, II and III of the GMFCS. The treadmill training had to be adapted to the tDCS procedures reported in the literature. The protocol described by Grecco *et al*. [9] was used as the basis for the present investigation. However, this protocol involves two weekly sessions of training over a seven-week period (total of 14 sessions). In the present study, five weekly sessions were held over a two-week period (total of 10 sessions). Thus, it was important to carry out a randomized controlled study involving a control group with placebo tDCS to determine the effects of treadmill training alone.

In a study involving patients with hemiparesis following a stroke, three sessions of anodal stimulation over the damaged motor cortex combined with specific training for the ankle of the paretic limb led to improvements in dorsiflexion and plantar flexion. This is in agreement with the present findings, as the strategies used by the ankle are fundamental to postural control and balance [27]. Another interesting study carried out by Kashi *et al.* (2013) [28] demonstrated that a single session of anodal stimulation in combination with balance and gait training resulted in improvements in balance, gait velocity and stride length in elderly individuals with leukoaraiosis (cerebral white matter lesion that affects gait and balance). In the present study, 10 consecutive sessions of tDCS were performed with the aim of potentiating the neuroplastic changes that occur from the combination of tDCS and motor training to determine whether the effects are persistent modifications of synaptic efficiency similar to long-term potentiation [29].

Kashi *et al.* (2012) [30] evaluated 30 healthy volunteers who received 15 minutes of anodal stimulation (2 mA; either active or placebo) of the prefrontal cortex while at rest prior to walking on a moving platform. The active group demonstrated improvements in postural control and gait velocity in comparison to the placebo group. These findings demonstrate that anodal tDCS is capable of causing changes in motor cortex excitability, thereby favoring motor control and lower limb movements.

In the present study, both groups demonstrated positive results following the different protocols. However, statistically significant differences between groups were found, with better results in the experimental group regarding anteroposterior sway, mediolateral sway and functional balance (PBS). These findings suggest that anodal stimulation of the primary motor cortex potentiated the results of treadmill training. The randomized, controlled study design allows the determination of the effect size, demonstrating the statistically significant effect of tDCS. One of the most important findings regards the fact that tDCS contributed to the maintenance of the effects of treadmill training. In clinical practice, the effects of physical therapy are often minimized or even completely lost following the interruption of the therapy sessions. In the present study, the gains achieved with the combination of treadmill training and tDCS remained one month after the completion of the protocol, suggesting the potential of tDCS to modify cortex excitability and favor neuroplasticity. The lack of an analysis of cortex excitability constitutes a limitation of this study. Although the aim was to analyze motor results, the measure of excitability could have allowed a more adequate explanation of the findings.

The possible adverse effects of tDCS should be addressed. However, the literature on tDCS in children is scarce and no previous papers involving motor training are found. In the present study, the children and their caregivers were asked about side effects at the end of each session and during the evaluations after the completion of the protocol. Three children in the experimental group experienced redness in the supra-orbital region (site of the cathode). No other adverse effects were reported, such as behavioral changes, headache or discomfort. During the sessions, 18 children (12 in the experimental group and 6 in the control group) reported a tingling sensation at the beginning of stimulation, but this sensation either ceased after a few seconds or was not considered bothersome. No children needed the intensity to be diminished or the stimulation to be stopped prior to the end of the 20-minute session. No children had difficulty performing treadmill training with tDCS and neither the wires nor the positioning of the electrodes hampered walking.

According to Kashi *et al*. (2012) [30], anodal tDCS induces changes in the excitability of the motor cortex referring to the lower limbs, with a consequent improvement in gait. Minhas *et al.* (2012) [26] carried out studies involving the administration of tDCS to children and found that the method is safe, but the current needs to be adjusted from 2 mA, which is used for adults, to 1 mA for children.

As a relatively new technique, few studies have employed tDCS on children with CP. Findings reported in the literature regard the use of transcranial magnetic stimulation as a method for analyzing the evoked potential [31] and cortex map [15]. This method has also been used to reduce spasticity in children with CP [32] in one or both hemispheres [15].

No studies were found addressing the effects of anodal tDCS over M1 on motor function or the combined use of tDCS and physical therapy, such as during gait training. Moreover, a limited number of studies discuss important clinical differences in CP, especially with regard to body sway and functional independence. For the present study, the findings described by Grecco *et al.* (2013) [20], who used the same treadmill training protocol, were considered clinically relevant.

The authors found no consensus in the literature or studies that specifically address a minimum difference that could be considered clinically important in the population with CP. However, as the present study involved a short intervention (2 weeks), the positive effect regarding the variables analyzed in the experimental group (gait training combined with anodal tDCS) at the follow up evaluation can be considered clinically important. The positive change in the PBS score in the experimental group allows one to infer that the quality of movement was optimized in these children. Indeed, the results demonstrate the effect size on functional balance in the experimental group vs. the control group at Evaluation 2 (5.6) and Evaluation 3 (5.2).

The findings of the present study demonstrate that the combination of treadmill training and anodal stimulation of the primary motor cortex in the dominant hemisphere was capable of potentiating improvements in static and functional balance in the children with cerebral palsy analyzed. Moreover, anodal stimulation favored the maintenance of the gains one month following the completion of the intervention. However, as this was a phase 2 study with a small sample size, further investigations with a larger number of participants and longer follow-up period are needed to confirm the results.

## Supporting Information

Checklist S1 **CONSORT checklist.**
(DOC)

Protocol S1 **Trial Protocol.**
(PDF)
