# Supplementary material for: Effect of Transcranial Direct-Current Stimulation Combined with Treadmill Training on Balance and Functional Performance in Children with Cerebral Palsy: A Double-Blind Randomized Controlled Trial
Source: PLoS One. 2014 Aug 29;9(8):e105777. doi: 10.1371/journal.pone.0105777 (PMC4149519; doi:10.1371/journal.pone.0105777)
Supplement: Protocol S1 — Trial Protocol. (PDF) [file pone.0105777.s002.pdf]

Effect of transcranial direct current stimulation combined with gait and mobility training on functionality in children with cerebral palsy: A double-blind randomized controlled clinical trial

Luanda André Collange Grecco[1§], Natália de Almeida Carvalho Duarte[1], Roberta Delasta Lazzari[1], Hugo Pasini[1], Leandro Henrique Grecco[2], Mariana de Mendonça[3], Lilian Chrystiane Giannasi Marson[4], Afonso Shiguemi Inoue Salgado[5], Renata Calhes Franco[6], João Carlos Ferrari Corrêa[1], Nelci Zanon Collange[7], Luciana Maria Malosá Sampaio[1], Felipe Fregni[8], Claudia Santos Oliveira[1§]

[1] Master's and Doctoral Programs in Rehabilitation Sciences, Nove de Julho University, São Paulo, Brazil.

[2]Master's Program in Pharmacology, Universidade de São Paulo, São Paulo, Brazil.

[3]Doctoral Program, Universidade de São Paulo, São Paulo, Brazil.

[4]Postdoctoral Fellowship, Postgraduate Program in Oral Biopathology, School of Dentistry, Universidade Estadual Paulista, São Paulo, Brazil.

[5]Physical Therapist, Doctoral student, Biomedical Engineering, Universidade Camilo Castelo Branco, São Paulo, Brazil.

[6]Professor of Physical Therapy, Universidade Nove de Julho, São Paulo, Brazil.

[7]Pediatric Neurosurgery, University Federal de São Paulo, São Paulo, Brasil.

[8]

[§]Corresponding author

Email Addresses:

LACG: luandacollange@hotmail.com

NACD: natycarvalho_fisio@hotmail.com

RDL: robertalazzari@hotmail.com

HP: hugopneto@yahoo.com.br

LHG: leandrohgrecco@hotmail.com

MM: mariana.fst@hotmail.com

LCGM: odontogiannasi@uol.com.br

ASIS: afonsoisalgado@yahoo.com.br

RCF: renatacalhes@uninove.br

JCFC: jcorrea@uninove.br

NZC: nelcizanon@terra.com.br

LMMS:lucianamalosa@uninove.br

FF:

CSO: csantos@uninove.br

Abstract

Background: The present project proposes three innovative intervention techniques (treadmill training, mobility training with virtual reality and transcranial direct current stimulation [tDCS]) that can be safely administered to children with cerebral palsy (CP). The combination of tDCS and physical therapy resources will provide the training of a specific task with multiple rhythmic repetitions of the phases of the gait cycle, providing rich sensory stimuli with a modified excitability threshold of the primary motor cortex to enhance local synaptic efficacy and potentiate motor learning.

Methods/Design: A prospective, double-blind, randomized, controlled, analytical, clinical trial will be carried out. Eligible participants will be children with CP classified on levels I, II and III of the Gross Motor Function Classification System (GMFCS) between four and ten years of age. The participants will be randomly allocated to four groups: 1) gait training on a treadmill with placebo tDCS; 2) gait training on a treadmill with active tDCS; 3) mobility training with virtual reality and placebo tDCS; and 4) mobility training with virtual reality and active tDCS. Transcranial direct current stimulation will be applied with the anodal electrode positioned in the region of the dominant hemisphere over C3, corresponding to the primary motor cortex, and the cathode positioned in the supraorbital region contralateral to the anode. A 1 mA current will be applied for 20 minutes. Treadmill training and mobility training with virtual reality will be performed in 30-minute sessions five times a week for two weeks (total of 10 sessions). Evaluations will be performed on four occasions: one week prior to the intervention; one week following the intervention; one month after the end of the intervention; and three months after the end of the intervention. The evaluations will involve three-dimensional gait analysis, analysis of cortex excitability (motor threshold and motor evoked potential), Six-Minute Walk Test, Timed

Up-and-Go Test, Pediatric Evaluation Disability Inventory, Gross Motor Function Measure, Berg Balance Scale, stabilometry, maximum respiratory pressure and an effort test.

Discussion: This paper offers a detailed description of a prospective, double-blind, randomized, controlled, analytical, clinical trial aimed at demonstrating the effect combining tDCS with treadmill and mobility training on functionality and primary cortex excitability in children with CP classified on GMFCS levels I, II and III. The results will be published and will contribute to evidence regarding the use of treadmill training on this population.

Trial Registration:

Background

Cerebral palsy (CP) refers to permanent, mutable motor development disorders stemming from a primary brain lesion, causing secondary musculoskeletal problems and limitations in activities of daily living [1]. The prevalence of CP ranges from 1.5 to 2.5 per 1000 live births, with little or no differences among Western nations [2]. Motor impairment is the main manifestation of this disease, with repercussions regarding the biomechanics of the body [3-4].

The concept of functional mobility regards how an individual moves through his/her environment for successful daily interactions with family and society [5] and is an important goal in the rehabilitation of children with CP. Walking with or without assistance allows such children greater participation in activities of daily living as well as better physical development [6]. Ninety percent of children with CP have impaired gait due to excessive muscle weakness, altered joint kinematics and diminished postural reactions [7]. Thus, such children have a diminished capacity for participating in games and sport activities at a sufficient intensity to develop an adequate degree of cardiopulmonary fitness [8-9].

Exercise programs that include aerobic and muscle strengthening components have often been contraindicated for children with CP due to the belief that greater effort during exercise would result in an increase in muscle tone, along with a reduction in the gamut of movements and global function [10-11]. However, a systematic review published in 2008 [11] reports evidence of the physiological benefits of aerobic exercise in children with CP, but the effect of these benefits on function remains unknown.

A number of approaches have been used to favor selective muscle control, coordinated muscle action during gait [7,12] and physical fitness [10,11]. Two such approaches are treadmill training and mobility and balance training with the aid of virtual reality techniques.

The development of new therapeutic resources for use in combination with physical rehabilitation methods is of fundamental importance to the optimization of the functional outcome [13]. Noninvasive cerebral stimulation has generated considerable interest in this

context, as significant functional improvement has been demonstrated following short periods of cerebral stimulation in individuals with brain lesions [13-14]. Transcranial Direct Current Stimulation (tDCS) is a promising method involving low-cost equipment that is easy to administer and offers good patient tolerance with minimum adverse effects [15]. tDCS has been used in combination with physical therapy to potentiate neuroplastic changes [13].

Treadmill training

In the last ten years, gait training on a treadmill has been used in the treatment of children with CP to optimize standing posture as well as improve gait speed and endurance. Although research in this field is still in the incipient stage, encouraging results have been demonstrated in children of different ages and different degrees of gross motor skill [14-22]. A treadmill can be used with or without partial weight support (PWS) to provide the training of a specific task with multiple repetitions of the steps of the gait cycle [21]. This method activates central pattern generators (CPGs) in lumbar region of the spinal cord [23]. CPGs are neural activations capable of forming motor patterns for the establishment of rhythmic, automatic steps, allowing the training of the biomechanical components of gait, postural control and balance [24-25]. MacKay-Lyons [26] raised the hypothesis that children with CP have impaired CPGs. The activation of these generators and mechanisms of automatic reciprocation is an important aspect of the stimulation of gait through treadmill training [20].

Systematic reviews of the literature [27-32] published in the last four years stress the small number of randomized, controlled, clinical trials of adequate methodological quality on this subject. The studies analyzed generally evaluate the effects of treadmill training with PWS involving heterogeneous samples in terms of functional level, as determined by the Gross Motor Function Classification System (GMFCS, levels I to IV) [33-34], and a relatively small number of participants. These reviews are categorical in stating that this form of intervention is safe, but

the studies offer divergent results and further investigations are needed to determine the benefits of treadmill training on children with CP.

The equipment available for PWS is costly and a specific infrastructure is often needed for the installation of this equipment, which limits its use in the home environment and even in the therapeutic setting. Thus, a large number of physical therapists opt for treadmill training without PWS in children with mild (GMFCS I-II) [33-34] to moderate (GMFCS III) [33-34] motor impairment. However, a discerning evaluation of the effects of this form of intervention on functional aspects is needed. Moreover, few studies have addressed ways of determining effective therapeutic parameters (duration, frequency and intensity of training). In a recent study carried out by Grecco *et al.* (2012) [35], treadmill training without PWS at a speed determined by the results of an effort test (at the aerobic threshold) demonstrated better results in comparison to overground gait training with regard to functional mobility (Six-Minute Walk Test and Timed Up-and-Go Test), gross motor function (walking, running and jumping), functional balance, static balance and cardiopulmonary fitness.

Training with virtual reality

Virtual reality is defined as an immersive, interactive, three-dimensional experience that occurs in real time [36-37], allowing the user to have a multidimensional, multisensory experience in a virtual environment [37-39]. The use of video games with a virtual reality device has been gaining ground in the rehabilitation process, especially in physical therapy. Researchers and clinicians have explored the use of Nintendo Wii^{TM} games as a rehabilitation tool for individuals with different forms of motor impairment involving deficits in balance and functional mobility [40]. *Exergames* is a relatively new term used to describe interactive electronic games that characterize the movements of the player as would occur in real life during the practice of a given exercise [41]. The Nintendo Wii program is a new style of virtual reality using either a remote control or wireless platform that allows the individual to interact with the representation

on the video screen through the use of a motion detection system. A sensor positioned on the television captures and reproduces the movement on the screen. The feedback provided by the image generates positive reinforcement, thereby facilitating the practice and perfection of the exercises. The games involve exercises of balance, functional mobility and aerobics [41].

It is believed that improvements can be achieved in the response to treatment through the play stimulus, adding a motivational factor to conventional treatment with the adoption of a specific game that motivates the patient to perform the desired movements [42-43]. The practical advantages of the use of virtual reality through Nintendo Wii™ regard the possibility of providing feedback in real time on the performance and progression of the exercise [44] and the ability to train at home with or without supervision [45]. Moreover, the system is a pleasant resource that can be used with family and friends [44].

However, few studies have investigated the use of e*xergames* in the realm of child neuromotor rehabilitation. Most studies involve adults and analyze the effects of balance in patients with sequelae stemming from a stroke [46] or sedentary, obese individuals [47]. By stimulating the displacement from the center of body mass and alterations in the support base, the games facilitate improvements in both static and dynamic balance during functional tasks [48]. Another benefit demonstrated in the literature regards the possibility of using the games as an alternative for the performance of aerobic exercises, promoting an improvement in physical fitness, according to the guidelines of the American College of Sports Medicine [49-50].

Practical guidelines for the use of virtual reality in the treatment of children with CP were published in February 2012 [51]. According to the manuscript, there is evidence to affirm that virtual reality is a promising tool in the treatment of such children. Despite the small number of studies carried out on this population, the findings demonstrate improvements in postural control, balance, upper limb function, selective motor control and gait [51].

Transcranial direct current stimulation

Transcranial direct current stimulation (tDCS) is a noninvasive method that stimulates the cerebral cortex by means of a direct, low-intensity, monophasic electric current (1 to 2mA) through surface electrodes. This method has advantages over other transcranial stimulation techniques, such as its ease of application, lower cost and more prolonged modulating effect on the cerebral cortex. Moreover, this type of intervention is better suited for comparison with placebo stimulation, thereby offering greater specificity in the results of a study [52-54].

The effects of tDCS are achieved through the movement of electrons due to the electrical charges between them. The anode pole of the electrode is positive and the cathode pole is negative. The electric current (electron flow) moves from the positive to the negative pole and has different effects on biological tissues. During the administration of tDCS, the electric current flows from the electrodes and penetrates the skull, reaching the cortex. Although a large portion of the current is dissipated among the overlying tissues, a sufficient amount reaches the structures of the cerebral cortex, altering the membrane potential of the surrounding cells [55-56].

tDCS has demonstrated effects on the excitability of the cerebral cortex. Short-term application has had short-lasting effects, whereas long-term application has generated long-lasting effects related to plastic mechanisms [57]. A large number of studies conducted on animal models report the polar effects of tDCS on the cerebral cortex, demonstrating that polarized currents applied to the brain surface may enhance spontaneous firing [58] and initiate paroxystic activity [59] when the anodal pole is used, whereas the cathode pole generally depresses these events. Based on these findings, studies involving humans have evaluated the effects of each pole on cortex excitability through the stimulation of the primary motor cortex, demonstrating that anodal and cathodal stimulation respectively enhances and diminishes cortex excitability [57].

tDCS is a neuromodulation technique that has piqued the interest of a large number of researchers in recent years. The results of clinical studies demonstrate the potential of this

method in the treatment of neurological conditions and investigations into the modulation of cerebral cortex excitability [54].

In the rehabilitation process, the aim of neuromodulation techniques is to enhance local synaptic efficacy by altering the maladaptive plasticity pattern that emerges following a cerebral cortex injury. The largest benefit of tDCS may come from its use in combination with different forms of physical therapy, as this method is a way to modulate the activity of the cerebral cortex, opening a path for the enhancement and prolongation of the functional gains provided by physical therapy. It can therefore be said that stimulation evokes a change in the dysfunctional excitability pattern so that physical therapy can model the functional pattern of cortex activity with the activation of specific neural networks [54].

Studies involving the use of tDCS on the primary motor cortex in stroke victims report improvements in upper limb function (active movements of wrists and fingers), movement velocity, active movements of the ankle and motor function. However, very few studies have analyzed the effects of tDCS on children with CP. Findings reported in the literature refer to Transcranial Magnetic Stimulation (TMS) as a way to analyze the evoked potential [60-62] and as a resource to reduce spasticity in children with CP [63]. A recent study investigating TMS found significant changes in the motor cortex maps of children with hemiparetic and diparetic CP (lateralization of upper limbs and motor representation of lower limbs), demonstrating the occurrence of reorganization following affections in one or both hemispheres of the brain [64].

The present project proposes three innovative intervention techniques (treadmill training, mobility training with virtual reality and transcranial direct current stimulation [tDCS]) that can be safely administered to children with cerebral palsy (CP). As CP results from a primary injury of the developing brain, with limitations in functional mobility in 90% of cases, it is reasonable to assume that the motor impairment found in patients stems from the combination of the brain lesion and maladaptive plasticity pattern that emerges following a cerebral cortex injury. Forms

of physical therapy seek to promote motor learning through the administration of functional training and multiple sensory stimuli. However, motor learning depends on a change in the excitability of the cerebral cortex, with a reduction in cortex inhibition following an injury. In this context, stimulation appears to be a way to modulate cortex activity, enhancing and extending the functional gains achieved with physical therapy [54].

Children with CP enter the rehabilitation process early and spend their entire lives performing a significant number of physical therapy sessions, which can have a negative effect on motivation. Thus, treadmill training and mobility training with the use of virtual reality offer such children a new therapy environment, which can pique their interest and enhance their motivation. Moreover, the combination of tDCS and physical therapy resources will provide the training of a specific task with multiple repetitions of the phases of the gait cycle, promoting rich sensory (proprioceptive and visual) stimuli with a modified threshold of excitability of the primary motor cortex (enhanced local synaptic efficacy), thereby potentiating motor learning.

Methods/Design

Primary objective

The primary aim of the proposed project is to perform a comparative analysis of the effects of treadmill training and mobility training with the use of virtual reality with and without tDCS on motor skills and cortex excitability in children with CP between the ages of four and ten years classified on levels I, II and III of the GMFCS.

Hypothesis 1

The combination of tDCS and either treadmill training or mobility training with virtual reality will achieve greater effects than the isolated use of these training modalities with regard to motor

skills and cortex excitability in children with CP between the ages of four and ten years classified on levels I, II and III of the GMFCS.

Study Design

A prospective, analytical, controlled, randomized, four-arm, double-blind study will be carried out (Figure 1). The protocol for this study is registered with the Brazilian Registry of Clinical Trials - ReBEC RBR-5v3kg9.

Insert Figure 1

Ethical considerations

The present study complies with the principles of the Declaration of Helsinki and the Regulating Norms and Directives for Research Involving Human Subjects formulated by the Brazilian National Health Council, Ministry of Health, established in October 1996. The study received approval from the ethics committee of the *Universidade Nove de Julho* (Sao Paulo, Brazil) under protocol number 69803/2012. The participating institutions have provided a declaration of participation. All guardians agreeing to the participation of their child will do so by signing a statement of informed consent. The participants will be allowed to abandon the study at any time with no negative repercussions.

Study sample and recruitment

Individuals with CP will be recruited from the physical therapy clinics of the *Universidade Nove de Julho* and *Centro de Neurocirurgia Pediátrica*, Sao Paulo, Brazil. The participants will be recruited and selected based on the following eligibility criteria:

Inclusion criteria

Age between four and 12 years

Cerebral palsy

Motor function classified as Level I, II or III by the GMCFS [6]

Levels 2 to 6 of the Functional Mobility Scale [18]

Independent ambulation with or without the need for a gait-assistance device (walker or crutches)

Exclusion criteria

Neurological or orthopedic conditions unrelated to cerebral palsy

Orthopedic surgery on the lower limbs in the 12 months prior to selection

Surgery scheduled during the period of the study

Orthopedic deformities with indication for surgery

Epilepsy

Metallic implant in skull or hearing aid

Sample size

The sample size was calculated with the aid of the STATA 11 program and based on a study carried out by Grecco *et al.* (2012) (*Effect of treadmill training without partial weight support on functionality in children with cerebral palsy: Randomized controlled clinical trial*). The Six-Minute Walk Test was considered for the calculation. This test was selected as the primary outcome based on its proven validity and reliability as a functional capacity assessment tool and will be used to evaluate the functional mobility and physical fitness of children with CP. Considering a mean and standard deviation of 377.2 ± 93.0 m in the experimental group and 268.0 ± 45.0 m in the control group, a bidirectional alpha of 0.05 and an 80% test power, eight children will be required for each group, to which 25% will be added to compensate for possible dropouts, totaling 40 participants (10 in each group).

Randomization

Following written agreement from parents/guardians to the participation of their children, those who meet the eligibility criteria will be randomly allocated to one of the four study groups using a block randomization method:

- Group 1: treadmill training with placebo tDCS;
- Group 2: treadmill training with active tDCS;
- Group 3: mobility training with virtual reality and placebo tDCS;
- Group 4: mobility training with virtual reality and active tDCS.

Randomization will be stratified based on the GMFCS (levels I-II and level III) and age (4-8 and 9-10 years). For each stratum, the allocation sequence will be determined using a randomization table. Following the pre-intervention evaluation, the participants will be allocated to the different groups based on the cards contained within sequentially numbered opaque envelopes. This process will be carried out by a member of the research team who is not involved in the recruitment process or development of the study.

Allocation concealment

A series of numbered, sealed, opaque envelopes will be used to ensure concealed allocation. Each envelope will contain a card stipulating to which group the child will be allocated.

Evaluation and follow-up

The children in both groups will be evaluated by two physical therapists experienced in the evaluation procedures and blinded to which group each child belongs. Four evaluations will be carried out:

Evaluation 1: one week prior to intervention;

Evaluation 2: one week following intervention;

Evaluation 3: one month following the end of intervention;

Evaluation 4: three months following the end of intervention.

Evaluations will be held on two non-consecutive days.

Functional mobility

The Six-Minute Walk Test [65-67] will be performed following the guidelines established by the American Thoracic Society [67]. This test quantifies functional mobility based on the distance in meters traveled in six minutes. During the test, the following physiological variables will be quantified: heart rate (HR), respiratory rate (RR), oxygen saturation ($SatO_2$), systolic blood pressure and diastolic blood pressure. Moreover, perceived respiratory and lower limb exertion will be determined using the Borg scale.

The Timed Up-and-Go Test is a fast, practical test that is widely employed to assess functional mobility and the risk of falls. This test quantifies functional mobility based on the time (in seconds) required for an individual to perform the task of standing up from a chair without arm supports, walking three meters, turning around, returning to the chair and sitting down again [68].

Three-dimensional gait analysis

Gait analysis will be performed with the aid of the SMART-D 140® system (BTS Engineering), involving the use of eight cameras sensitive to the infrared spectrum and the SMART-D INTEGRATED WORKSTATION® with 32 analogue channels. Two force plates (Kistler, model 9286BA) will be used for the kinetic gait data (displacement from the center of pressure and contact time of the foot with the surface of the platform). All children will be wearing bathing

suits to facilitate the placement of the markers. The skin will be cleaned with alcohol to allow the proper attachment of the markers on precise anatomic sites. Reflective markers will be placed on the skin using the biomechanical model described by Davis for the acquisition of kinematic gait data [69-70].

The participants will walk along a path marked on the floor measuring 90 centimeters in width and four meters in length, with the two force plates positioned in the center. Upon stepping onto the force plates, the kinetic gait data will be collected and calculated using a video system (BTS, Milan, Italy) synchronized with the kinematic data collection system. The electrical activity resulting from the activation of the rectus femoris, tibialis anterior and soleus muscles will be collected using an eight-channel electromyograph (FREEEMG® – BTS Engineering), containing a bioelectric signal amplifier, wireless data transmission and bipolar electrodes with a total gain of 2000 and 20-450 Hz sampling frequency. Impedance and the common rejection mode will be $>10^{15}$ Ω//0.2 pF and 60/10Hz 92 dB, respectively. The motor point of the muscles will be identified for the placement of the electrodes and the skin will be cleaned with 70% alcohol to reduce bioimpedance, as recommended by the Surface Electromyography for the Non-Invasive Assessment of Muscles (SENIAM) [71]. All electromyographic data will be collected and digitized a 1000 frames/second using the BTS MYOLAB® program. These data will be collected simultaneously with the kinematic and kinetic data and all data will be managed using the BTS® system and Smart Capture® program.

Cortex excitability

Transcranial magnetic stimulation will be employed for the evaluation of cortex excitability using a magnetic stimulator with a figure-eight coil (Magstin 200^2). Responses to the stimulus applied to the motor cortex will be recorded in the tibialis anterior muscle of the contralateral lower limb. The responses of the motor evoked potential (MEP) will be filtered and amplified using surface electromyography. The signals will be transferred to a personal computer for off-line analysis

using the data collection software program. Motor threshold and MEP measurements will be performed [72] using single-pulse TMS. The motor threshold will be found in the region of the cortex with the least intensity necessary to generate a peripheral response. The same method will be used to assess the MEP, using 110% of the intensity of the motor threshold. Ten MEP measurements will be performed in each step of the evaluation.

Functional Performance

The Pediatric Evaluation of Disability Inventory (PEDI) quantitatively measures functional performance. This questionnaire will be administered in interview form to one of the child's caregivers who has information on the performance of the child regarding typical activities and tasks of daily routine. The first part of the questionnaire will be used, which assesses skills in the child's repertoire grouped into three functional categories: self-care (73 items), mobility (59 items) and social function (65 items). Each item is scored 0 (zero) when the child is unable to performed the activity or 1 (one) when the activity is part of the child's repertoire of skills. The scores are totaled per category [73-75].

Gross Motor Function

The Gross Motor Function Measure (GMFM-66) allows a quantitative assessment of gross motor function in individuals with CP and has proven validity and reliability. The measure is made up of 66 items distributed among five subscales: A) lying down and rolling; B) sitting; C) crawling and kneeling; D) standing; and E) walking, running and jumping. The items of each subscale receive a score of 0 to 3 points, with higher scores denoting better performance [76-77].

Static Balance

The evaluation of static balance will be performed on a pressure plate (Kistler, model 9286BAO, which allows stabilometric analysis based on oscillations of the center of pressure (COP). The acquisition frequency will be 50 Hz, captured by four piezoelectric sensors measuring 400/600 mm positioned at the extremities of the platform. The data will be recorded and interpreted using the SWAY program (BTS Engineering), integrated and synchronized to the SMART-D 140® system. The children will be instructed to remain standing on the platform, barefoot, arms alongside the body, gazed fixed on a point marked at a distance of one meter at the height of the glabellum (adjusted for each child), with an unrestricted foot base and heels aligned. Readings will be made for 30 seconds each under two conditions (eyes open and eyes closed). Displacement from the COP on the X (anteroposterior) and Y (mediolateral) axes will be measured under both conditions [78].

Functional Balance

The Berg Balance Scale will be used for the assessment of functional balance. This simple 14-item measure addresses the performance of functional balance common to daily living. Each item has a five-option scale ranging from 0 to 4 points, with a maximum overall score of 56. The points are based on the time in which a position is maintained, the distance an upper limb is able to reach in front of the body and the time needed to complete the task. Total execution time is approximately 30 minutes. The children will perform these tasks dressed, but barefoot [79-80].

Respiratory muscle strength

Respiratory muscle strength will be determined based on maximum inspiratory pressure (IPmax) and expiratory pressure (EPmax), which respectively measure inspiratory and expiratory muscle strength and will be determined using the method described by Black & Hyatt [81]. For such, a gauge scaled in cmH_2O will be used, with an operational limit of ± 300 cmH_2O

and equipped with a mouth adapter and escape valve – an orifice approximately 2 mm in diameter to prevent a rise in pressure in the oral cavity generated exclusively by facial muscle contractions. IPmax will be determined by maximum inspiration beginning from maximum expiration and EPmax will be determined by maximum expiration beginning from maximum inspiration. Each maneuver will be maintained for at least two to three seconds. IPmax and EPmax will be determined at least three times for each child, with the largest value used in the analysis.

Treadmill test

There is no standardized test for the pediatric population with neurological disorders. The most often employed test in pediatrics is the modified Bruce protocol. However, this includes the inclination of the treadmill, which makes it extremely difficult for children with moderate to severe motor impairment. The proposed study will employ the symptom-limited cardiopulmonary effort test on a treadmill (Imbramed Mileniun ATL), using the ramp protocol with increasing speed (initially 0.5 km/h and increased 0.5 km/h each minute). The following will be the criteria for interrupting the test: subjective sensation of fatigue, lower limb pain reported by child, complex heart arrhythmia, sudden increase or drop in blood pressure (BP), increase above maximum HR predicted for age of the individual, intense shortness of breath and drop in oxygenation accompanied by electrocardiographic alterations or signs and symptoms. At each stage of the test, the child will be asked about shortness of breath and lower limb pain and the subjective responses will be classified using the Borg Perceived Exertion Scale. During the test, BP will be measured on the left arm with a portable sphygmomanometer and stethoscope (Diasist brand) using indirect auscultation. Electromyographic activity will be monitored using an Ecafix monitor and HR will be monitored with a Polar Electro Oy heart rate meter. Oxygen saturation will be monitored continuously during the treadmill test using a portable oximeter (Nonin 8500A).

A rest period will be granted between the administration of each measure and the children will be allowed to interrupt the evaluation at any moment to rest. Following a minimum period of 20 minutes of rest, HR and RR will be measured. The time between the administration of the assessment measures will be sufficient for these rates to return to resting values to ensure a sufficient rest period so that the child's performance is not compromised.

Intervention

Transcranial direct current stimulation

A neurologist with ample experience in noninvasive cerebral stimulation will be in charge of the evaluations for the indication of tDCS. tDCS will be performed during the intervention sessions, as this technique may facilitate behavioral changes through the establishment of a neural network favorable to the environment. For such, the tDCS Transcranial Stimulation equipment (Trans Cranial Technologies, USA) will be employed, using two non-metallic sponge surface electrodes measuring 5 x 5 cm^2 soaked in saline solution. The children will be randomly distributed into two types of treatment: 1) anodal stimulation of the primary motor cortex and 2) placebo tDCS.

The anodal electrode will be positioned in the region of the dominant brain hemisphere over C3, following the internationally standardized 10-20 electroencephalogram system, corresponding to the primary motor cortex [82], and the cathode will be positioned in the contralateral supraorbital region. Placebo stimulation will involve the placement of the electrodes and the stimulator will be switched on for 30 seconds to give the child an initial sensation. However, no stimulation will be offered throughout the rest of the session. This is a valid control procedure in studies involving tDCS.

The current will be applied to the primary motor cortex for 20 minutes in the middle of each session. The device has a button that allows the operator to control the intensity of the current. Stimulation will be raised from 0 to 1 mA and gradually diminished in the final ten seconds.

Gait and mobility training protocols

The training protocols will entail five weekly 30-minute sessions over two consecutive weeks (total of 10 sessions). During the training, the children in all groups use their own orthoses and habitual gait-assistance device, if necessary. A pre-intervention evaluation will be held to determine whether the gait-assistance device is of an adequate size for the child and make the necessary adjustments. The orthoses will be duly placed by the physiotherapist. HR will be monitored is during all sessions to ensure the non-occurrence of overload on the cardiovascular system.

Treadmill training: The Milenium ATL treadmill (Imbramed, RS, Brazil) will be used. Two treadmill training sessions will be held prior to the onset of the intervention to familiarize the children with the equipment. During these initial sessions, the children will not receive tDCS. The velocity will be gradually increased based on the child's tolerance. During the sessions, treadmill speed will be maintained at 60 to 80% of the maximum speed previously established on the exertion test, performed based on the method reported by Grecco *et al.* (2012).[35] The child will walk at 60% maximum speed in the first and final five minutes and 80% in the middle 20 minutes.

Mobility training with virtual reality: Nintendo Wii™ will be used with the Wii Fit Plus™ program and Wii Balance Board. The Wii Fit Plus™ package is made up of more than 50 games. For the study, however, a balance exercise (hula hoop) and two aerobic exercises (walking and walking with obstacles) will be used. The child will first be instructed to stand on

the Wii Balance Board for the estimate of height and calculation of the body mass index. Two sessions of mobility training with the Wii Fit Plus^{TM} exercises will be held prior to the protocol. During the training sessions, the first and final five minutes will be dedicated to the virtual hula hoop exercise and the middle 20 minutes will be dedicated to walking with and without obstacles. The training will be held in a specific room of the Movement Analysis Laboratory (*Universidade Nove de Julho*) measuring 250 X 400 cm, with a projection screen (200 X 150 cm) and stereo speakers attached to the wall to provide adequate visual and audio stimuli.

The number of sessions attended, maximum speed of treadmill training, duration of treadmill training and distance travelled in each session will be recorded on a follow-up chart. Any problems or injuries that may occur during training will also be recorded. All participants will be instructed to maintain their normal daily activities and attend normal physical therapy sessions, if undergoing such therapy.

Statistical analysis

The Kolmogorov-Smirnov test will be used to determine whether the data adhere to the Gaussian curve. Parametric data will be expressed as mean (standard deviation) and nonparametric data will be expressed as median (inter-quartile interval). The effect size will be calculated by the difference between means of the pre-intervention and post-intervention evaluations and will be presented with the respective 95% confidence interval. Either repeated-measures ANOVA or Friedman's test will be used for the statistical analysis of the immediate effects of tDCS for parametric and nonparametric variables, respectively. Either two-way ANOVA or the Kruskal-Wallis test will be used for the statistical analysis of the effects of mobility training with and without tDCS for parametric and nonparametric variables, respectively. All p-values < 0.05 will be considered significant. The data will be organized and tabulated using the Statistical Package for the Social Sciences (SPSS v.19.0).

Discussion

This paper offers a detailed description of a randomized, controlled, blinded, clinical trial aimed at demonstrating the effects of the combination of tDCS and either treadmill training or mobility training on functionality and cortex excitability in children with CP classified on GMCS levels I, II and III. The results will be published and will contribute evidence regarding the use of tDCS and treadmill training on this population.

List of abbreviations

BP: Blood Pressure;

Cm: Centimeter;

CmH_2O: Centimeter of Water;

CP: Cerebral Palsy;

COP: Center of Pressure;

dB: Decibels;

EPmax: Maximum Expiratory Pressure;

FAPESP: Fundação de Amparo á Pesquisa do Estado de São Paulo;

GMFCS: Gross Motor Function Classification System;

GMFM: Gross Motor Function Measure;

HR: Heart Rate;

Hz: Hertz;

IPmax: Maximum Inspiratory Pressure;

Km/h: Kilometer per hour;

mA: Milliampere;

MEP: Motor Evoked Potential;

MM: Millimeter;

PEDI: Pediatric Evaluation of Disability Inventory;

REBEC: Brazilian Registry of Clinical Trials;

RR: Respiratory Rate;

SatO2: Oxygen Saturation;

SENIAM: Surface Electromyography for the Non-Invasive Assessment of Muscles;

SPSS: Statistical Package for the Social Sciences;

tDCS: Transcranial Direct Current Stimulation;

TMS: Transcranial Magnetic Stimulation;

Competing interests

The authors declare that they have no competing interests.

Funding

This protocol study received funding from the *Fundação de Amparo á Pesquisa do Estado de São Paulo* (FAPESP). The main investigator receives monthly financing in the amount of twelve hundred Reals (Brazilian currency) from the Brazilian government agency FAPESP for the development of the study protocol.

Ethical considerations

The present study complies with the principles of the Declaration of Helsinki and the Regulating Norms and Directives for Research Involving Human Subjects formulated by the Brazilian National Health Council, Ministry of Health, established in October 1996. The study received approval from the ethics committee of the *Universidade Nove de Julho* (Sao Paulo, Brazil) under protocol number 69803/2012.

Authors' contributions

All authors contributed to the conception and design of the study. CSO and LACG provided the idea for the study, established the hypothesis and wrote the original proposal. LACG and CSO significantly contributed to the drafting of this paper, while HP, LHG, RDL, NACD, LCGM, ASIS, RCF, JCFC, NZC and LMMS were involved in critically revising the manuscript. This protocol paper was written by LACG and CSO with input from all co-authors. All authors read and approved the final manuscript.

Acknowledgments and funding

The Integrated Movement Analysis Laboratory receives funding from the *Universidade Nove de Julho* (Brazil) and research projects approved by the Brazilian fostering agency *Fundação de Amparo á Pesquisa do Estado de São Paulo* (FAPESP).

References

1. Rosenbaum P, Paneth N, Leviton A, Goldstein M, Bax M: **A report: the definition and classification of cerebral palsy.** *Dev Med Child Neurol* 2007, **49**(s109):8-14.
2. Paneth N, Hong T, Korzeniewski S: **The descriptive epidemiology of cerebral palsy.** *Clin Perinatol* 2006, **33**(2)251-67.

3. Kavcic A, Vodusek BD: **A historical perspective on cerebral palsy as a concept and a diagnosis.** *Eur J Neurol* 2005, **12**(8):582-7.
4. Awaad Y, Taynen H, Munoz S, Ham S, Michon AM, Awaad R: **Functional assessment following intrathecal baclofen therapy in children with spastic cerebral palsy.** *J Child Neurol* 2003, **18**(1):26-34.
5. Organização Mundial de Saúde, Organização Panamericana da saúde: **Classificação Internacional de Funcionalidade, Incapacidade e Saúde.** São Paulo: Editora da Universidade de São Paulo; 2003.
6. Mattern-Baxter K, Bellamy S, Mansoor JK: **Effects of intensive locomotor treadmill training on young children with cerebral palsy.** *Pediatric Phys Ther* 2009, **21**:308-19.
7. Chagas PSC, Mancini MC, Barbosa A, Silva PTG: **Análise das intervenções utilizadas para a promoção da marcha em crianças portadoras de paralisia cerebral: uma revisão sistemática da literatura.** *Rev Bras Fisioter* 2004, **8**(2):155-63.
8. Bjornson KF, Belza B, Kartin D, Logsdon R, McLaughlin JF: **Ambulatory physical activity performance in youth with cerebral palsy and youth who are developing typically.** *Phys Ther* 2007, **87**: 248-57.
9. Fowler EG, Knutson LM, Demuth SK, Sieber KL, Simms VD, Sugi MH, Souza RB, Karin E, Azen SP: **Pediatric endurance and limb strengthening (PEDALS) for children with cerebral palsy using stationary cycling: a randomized controlled trial.** *Phys Ther* 2010, **90**(3):367-81.
10. Dodd KJ, Taylor NF, Damiano DL: **A systematic review of the effectiveness of strength-training programs for people with cerebral palsy.** *Arch Phys Med Rehabil* 2002, **83**:1157-64.
11. Roger A, Furler BL, Brinks S, Darrah J: **A systematic review of the effectiness of aerobic exercise interventions for children with cerebral palsy: an AACPDM evidence report.** *Dev Med Child Neurol* 2008, **50**(11):808-11.

12. Silva MS, Daltrário SMB: **Paralisia cerebral: desempenho funcional após treinamento da macrha em esteira.** *Fisioter Mov* 2008, **21**(3):109-15.
13. Stagg CJ, Bachtiar V, O'Shea J, Allman C, Bosnell RA, Kischka U, Matthews PM, Johansen-Berg H: **Cortical activation changes underlying stimulation induced behavioral gains in chronic stroke.** *Brain* 2012, **135**:276-84.
14. Hummel F, Cohen L: **Non-invasive brain stimulation: A new strategy to improve neurorehabilitation after stroke?** *Lancet Neurol* 2006, **5**:708-12.
15. Smania N, Bonetti P, Gandolfi M et al: **Improved gait after repetitive locomotor training in children with cerebral palsy.** *Am J Phys Med Rehabil* 2011, **90**:137-49.
16. Richards CL, Malouin F, Dumas F, Marcoux S, Lepage C, Menier C: **Early and intensive treadmill locomotor training for young children with cerebral palsy: A feasibility study.** *Pediatric Phys Ther* 1997, **9**(4):159-65.
17. Cherng R, Liu C, Lau T, Hong R: **Effect of treadmill training with body weight support on gait and gross motor function in children with spastic cerebral palsy.** *Am J Phys Med Rehabil* 2007, **86**(7):548-55.
18. Dodd KJ, Foley S: **Partial body-weight–supported treadmill training can improve walking in children with cerebral palsy: A clinical controlled trial.** *Dev Med Child Neurol* 2007, **49**(2):101-5.
19. Verschuren O, Ketelaar M, Gorter JW, Helders PJ, Uiterwaal CS, Takken T: **Exercise training program in children and adolescents with cerebral palsy: a randomized controlled trial.** *Arch Pediatr Adolesc Med* 2007, **161**(11):1075-81.
20. Willoughby KL, Dodd KJ, Shields N, Foley S. **Efficacy of partial body weight–supported treadmill training compared with over ground walking practice for**

children with cerebral palsy: a randomized controlled trial. *Arch Phys Med Rehabil* 2010, **91**(3):333-9.

21. Mattern-Baxter K: **Effects of partial body weight supported treadmill training on children with cerebral palsy.** 2009, **21**:12-22.
22. Johnston TE, Watson KE, Ross SA et al: **Effects of a supported speed treadmill training exercise program on impairment and function for children with cerebral palsy**. *Dev Med Child Neurol* 2011, **53**(8):742-50.
23. Dimitrijevic MR, Gerasimenko Y, Pinter MM: **Evidence for a spinal central pattern generator in humans.** *Annals of the New York Academy of Sciences* 1998, **16**(860):360–76.
24. Barbeau H: **Locomotor training in neurorehabilitation: emerging rehabilitation concepts.** *Neurorehabilitation and Neural Repair* 2003, **17**:3-11.
25. Louza CM, Macedo LB: **Gerador de padrão central da locomoção humana: uma visão neurofuncional e prática na reabilitação.** *Arquivos Brasileiros de Paralisia Cerebral* 2005, **1**(2):4-10.
26. Mackay-Lyons M: **Central pattern generation of locomotion: A review of the evidence.** *Physical Therapy* 2002, **82**(1):69–83.
27. Mattern-Baxter K: **Locomotor treadmill training for children with cerebral palsy**. *Orthopaedic Nursing* 2010, **29**(3):169-73.
28. Damiano D, Dejong S: **A systematic review of the effectiveness of treadmill training and body weight support in pediatric rehabilitation**. *J Neurol Phys Ther* 2009, **33**:27-44.
29. Mutlu A, Krosschell K, Spira DG: **Treadmill training with partial body-weight support in children with cerebral palsy: a systematic review.** *Dev Med Child Neurol* 2009, **51**(4):268-75.

30. Molina-Rueda F, Aguila-Maturana AM, Molina-Rueda MJ, Miangolarra-Page JC: **Treadmill training with or without partial body weight support in children with cerebral palsy: systematic review and meta-analysis.** *Rev Neurol* 2010, **51**(3):135-45.

31. Zwicker JG, Mayson TA: **Effectiveness of theadmill training in children with motor impairments: an overview of systematic reviews.** *Pediatr Phys Ther* 2010, **22**:361-77.

32. Grecco LAC, Pasini H, Sampaio LMM, Oliveira CS: **Evidence of effect of treadmill training on children with cerebral palsy: a systematic review.** *Clin Exp Med Lett* 2012 (artigo aceito).

33. Palisano R, Rosenbaum P, Walter S, Russel D, Wood E, Galuppi B: **Development and reliability of a system to classify gross motor function in children with cerebral palsy.** *Dev Med Child Neurol* 1997, **39**(4):214-23.

34. Hiratuka E, Matsukura TS, Pfeifer LI: **Cross-cultural adaptation of the Gross Motor Function Classification System into Brazilian-Portuguese (GMFCS).** *Rev Bras Fisioter* 2010, **14**(6):537-44.

35. Grecco LAC, Pasini H, Costa IP, Zanon N, Sampaio LMM, Oliveira CS: **Effect of treadmill training on physical fitness and fucntional performance of children with cerebral palsy: preliminary study, single-blind, randomized controlled trial.** *Physicotherapy* 2012 (artigo aceito).

36. Reid DT: **The influence of virtual reality on playfulness in children with cerebral palsy: a pilot study.** *Occup Ther* 2004, **11**:131-144.

37. Deutsch JE, Borbely M, Filler J, Huhn K, Guarrera-Bowlby P: **Use of a low-cost, commercially available gaming console (Wii) for rehabilitation of an adolescent with cerebral palsy.** *Phys Ther* 2008, **88**:1196-1207.

38. Weiss P, Rand D, Katz N, Kizony R: **Video capture virtual reality as a flexible and effective rehabilitation tool. J.** *Neuroeng. Rehabil* 2004, **1**:12.
39. Sveistrup H: **Motor rehabilitation using virtual reality: review.** *J. Neuroeng. Rehabil* 2004, **1**:10-18.
40. Lange B, Flynn S, Proffitt R, Chang CY, Rizzo AS: **Development of an interactive game based rehabilitation tool for dynamic balance training.** *Top Stroke Rehabil* 2010, **17**(5):345-52.
41. Bailey BW, McInnis K: **Energy cost of exergaming: a comparison of the energy cost of 6 forms of exergaming.** *Arch Pediatr Adolesc Med* 2011, **165**(7):597-602.
42. Abdalla TCR, Prudente COM, Ribeiro MFM, Souza JS. **Analysis of the evolution of standing balance in children with cerebral palsy under virtual rehabilitation, aquatic therapy and physiotherapy traditional**. *Rev Mov* 2010, **3**(4):181-86.
43. Clark RA, Bryant AL, Yonghao P, McCrory P, Bennell K, Hunt M: **Validity and reliability of the Nintendo Wii Balance Board for assessment os standing balance.** *Gait Posture* 2010, **31**(3):307-10.
44. Hurkmans HL, Ribbers GM, Streur-Kranenburg MF, Stam HJ, van den Berg-Emons RJ: **Energy expenditure in chronic stroke patients playing Wii Sports: a pilot study.** *J Neuroeng Rehabil* 2011, **14**:8-38.
45. Rizzo A, Kim GJ: **A SWOT analysis of the field of virtual reality rehabilitation and therapy.** Presence: *Teleoper Virtual Environ* 2005, **14**:119-46.
46. Barcala L, Colella F, Araujo MC, Salgado ASI, Oliveira CS: **Balance analysis in hemiparetics patients after training with Wii Fit program.** *Fisioter Mov* 2011, **24**(2):337-43.

47. Garn AC, Baker BL, Beasley EK, Solmon MA: **What are the benefits of a commercial exergaming platform for college students? Examining physical activity, enjoyment, and future intentions.** *J Phys Act Health* 2012, **9**(2):311-8.
48. Duclos C, Miéville C, Gagnon D, Leclerc C. **Dynamic stability requirements during gait and standing exergames on the Wii Fit system in the elderly.** *J. Neuroeng Rehabil* 2012, **9**:28.
49. Guderian B, Borreson LA, Sletten LE, Cable K, Stecker TP, Probst MA, Dalleck LA: **The cardiovascular and metabolic responses to Wii Fit video game playing in middle-aged and older adults.** *J Sports Med Phys Fitness* 2010, **50**(4):436-42.
50. Douris PC, McDonald B, Vespi F, Kelley NC, Herman L: **Comparison between Nintendo Wii Fit aerobics and traditional aerobic exercise in sedentary young adults.** *J Strength Cond Res* 2012, **26**(4):1052-7
51. Pereira E, Rueda MF, Diego A, Cuerda CG, De Mauro A, Page MJC: **Use of virtual reality systems as proprioception method in cerebral palsy: clinical practice guideline.** *Neurologia* 2012, 16.
52. Fregni F, Gimenes R, Valle AC, Ferreira MJ, Rocha RR, Natalle L, Bravo R, Rigonatti SP, Freedman S, Nitsche M, Pascual-Leone A, Boggio OS: **A randomized, sham-controlled, proof of principle study of transcranial direct current stimulation for the treatment of pain in fibromyalgia.** *Arthritis and Rheumatism* 2006, **54**:3988-98.
53. Fregni F, Bossio PS, Brunoni AR: Neuromodulação terapêutica: Princípios e avanços da estimulação cerebral não invasiva em neurologia, reabilitação, psiquiatria e neuropsicologia. Sarvier. São Paulo; 2012.
54. Mendonça ME, Fregni F: **Neuromodulação com estimulação cerebral não invasiva: aplicação no acidente vascular encefálico, doença de Parkinson e dor crônica.** In.:ASSIS, R.D. **Condutas práticas em fisioterapia neurológica.** Manole. São Paulo, p. 307-39, 2012.

55. Miranda PC, Lomarev M, Hallett M: **Modeling the current distribution during transcranial direct current stimulation.** *Clin Neurophysiol* 2006, **117**(7):1623-9.

56. Wagner T, Fregni F, Fecteau S, Grodzinsky A, Zahn M, Pascual-Leone A: **Transcranial direct current stimulation: A computer-based human model study.** *Neuroimage* 2007, 35:**1113**-24.

57. Nitsche MA, Paulus W: **Sustained excitability elevations induced by transcranial DC motor cortex stimulation in humans.** *Neurology* 2001, **27**(10):1899-901.

58. Creutzfeldt OD, Fromm GH, Kapp H: **Influence of transcortical d-c currents on cortical neuronal activity.** *Exp Neurol* 1962, **5**:436-52.

59. Goldring S, O'Leary JL: **Summation of certain enduring sequelae of cortical activation in the rabbit.** *Electroencephalogr Clin Neurophysiol* 1951, **3**(3):329-40.

60. Nezu A, Kimura S, Takeshita S, Tanaka M: **Functional recovery in hemiplegic cerebral palsy: ipsilateral electromyographic responses to focal transcranial magnetic stimulation**. *Brain Dev* 1999, **21**(3):162-5.

61. Garvey MA, Mall V: **Transcranial magnetic stimulation in children**. *Clin. Neurophysiol* 2008, **119**(5):973-84.

62. Vry J, Linder-Lucht M, Berweck S, Bonati U, Hodapp M, Uhi M, Faist M, Mall V: **Altered cortical inhibitory function in children with spastic diplegia: a TMS study.** *Exp. Brain Res* 2008, **186**(4):611-8.

63. Valle AC, Dionisio K, Pitskel NB, Pascual-Leone A, Orsati F, Ferreira MJ, Boggio PS, Lima MC, Rigonatti SP, Fregni F: **Low and high frequency repetitive transcranial magnetic stimulation for the treatment of spasticity.** *Dev. Med. Child. Neurol* 2007, **49**(7):534-8.

64. Kesar TM, Sawaki L, Burdette JH, Cabrera MN, Kolaski K, Smith BP, O'Shea TM, Koman LA, Wittenberg GF: **Motor cortical functional geometry in cerebral palsy and its relationship to disability.** *Clin Neurophysiol* 2012, **123**(7):1383-90.

65. Li AM, Yin J, Yu CCW, Tsang T So HK, Wong E, Chan D, Hon EK, Sung R: **The six minute walk test in healthy children: reliability and validity**. **Eur Respir J** v. 25, p. 1057–60, 2005.

66. Maher CA, Williams MTA, Olds T: **The six-minute walk test for children cerebral palsy**. *Int J Rehabil Res* 2008, **31**(2):185-8.

67. American Thoracic Society: **ATS statement: guidelines for the six-minute walking test. Committee on Profiency Standards for Clinical Pulmonary Function Laboratories.** *Am J Respir Crit Care Med* 2002, **166**:111-17.

68. Williams LN, Carroll SG, Reddihough DS, Phillips BA, Gallea BA, Galea MP: **Investigation of the timed 'Up & Go' test in children**. *Dev Med Child Neurol* 2005, **47**:518–24.

69. Davis RB, Ounpuu S, Tyburski D, Gage JR: **A gait analysis data collection and reduction technique.** *Hum Mov Sci* 1991, **10**(5): 575–587.

70. Kadaba MP, Ramakrishnan HK, Wooten ME: **Measurement of lower extremity kinematics during level walking.** *J Orthop Res* 1990, **8**: 383-92.

71. Hermes JH, Freriks B, Merletti R, Steggeman D, Blok J, Rau G, Disselhorst-Klug C, Hagg G: **Surface Electromyography for the Non-Invasive Assessment of Muscles** 1999.

72. Rossini PM, Barker AT, Berardelli A *et al:* **Non-invasive electrical and magnetic stimulation of the brain, spinal cord and roots: Basic principles and procedures for routine clinical application.** *Electroencephalogr Clin Neurophysiol* 1994, **91**:79-992.

73. Feldman AB, Haley SM, Corvell J: **Concurrent andconstruct validity of the Pediatric Evaluation of Disability Inventory**. *Phys Ther* 1990, **70**(10):602-10.

74. Haley SM, Coster J, Faas RM: **A content validity study of the Pediatric Evaluation of**

Disability Inventory. *Pediatr Phys Ther* 1991, **3**:177-84.

75. Haley SM, Coster WJ, Ludlow LH, Haltiwanger JT, Andrelow PJ: **Inventário de avaliação pediátrica de disfunção: versão brasileira**. Tradução e adaptação cultural MC Mancini. Belo Horizonte: Laboratório de Atividade e Desenvolvimento Infantil, Departamento de Terapia Ocupacional, UFMG; 2000.
76. Russel DJ, Rosenbaum PL, Avery LM, Lane M: **Gross Motor Function Measure (GMFM-66 & GMFM-88) User's Manual**. London, UK: Mac Keith Press; 2002.
77. Russel DJ, Avery LM, Rosenbaum PL, Raina PS, Walter SD, Palisano RJ: **Improved scaling of the gross motor function measure for children with cerebral palsy: evidence of reliability and validity.** *Phys Ther* 2009, **80**(9):873-85.
78. Nobre A, Monteiro FF, Golin MO, Biasotto-Gonzalez D, Corrêa JC, Oliveira CS: **Analysis of postural oscillation in children with cerebral palsy.** *Electromyogr Clin Neurophysiol* 2010, **50**(5):239-44.
79. Berg K, Wood-Dauphinee S, Williams JI, Gayton D: **Measuring balance in the elderly: preliminary development of an instrument.** *Physiother Can* 1989, **41**:304–11.
80. Kembhavi G, Darrah J, Magill-Evans J, Loomis J: **Using the Berg Balance Scale to distinguish balance abilities in children with cerebral palsy.** *Pediatr Phys Ther* 2002, **14**:92–99.
81. Camelo JS, Terra Filho J, Manco JC: **Maximal respiratory pressures in normal adults.** *J pneumol* 1985, **11**(4):181-184.
82. Homan RW, Herman J, Purdy P: **Cerebral location of international 10-20 system electrode placement.** *Electroencephalogr Clin Neurophysiol* 1987, **66**(4):376-82.
